# Performance of Serum microRNAs -122, -192 and -21 as Biomarkers in Patients with Non-Alcoholic Steatohepatitis

**DOI:** 10.1371/journal.pone.0142661

**Published:** 2015-11-13

**Authors:** Philip P. Becker, Monika Rau, Johannes Schmitt, Carolin Malsch, Christian Hammer, Heike Bantel, Beat Müllhaupt, Andreas Geier

**Affiliations:** 1 Department of Gastroenterology and Hepatology, University Hospital Zürich (USZ), Zürich, Switzerland; 2 Division of Hepatology, University Hospital Würzburg (UKW), Würzburg, Germany; 3 Institute of Clinical Epidemiology und Biometry University of Würzburg, Würzburg, Germany; 4 School of Life Sciences, École Polytechnique Fédérale de Lausanne, Lausanne, Switzerland; 5 Department of Gastroenterology, Hepatology and Endocrinology, Hannover Medical School, Hannover, Germany; 6 Zürich Center for Integrative Human Physiology (ZIHP), University of Zürich (UZH), Zürich, Switzerland; University Hospital of Essen, GERMANY

## Abstract

**Objectives:**

Liver biopsies are the current gold standard in non-alcoholic steatohepatitis (NASH) diagnosis. Their invasive nature, however, still carries an increased risk for patients’ health. The development of non-invasive diagnostic tools to differentiate between bland steatosis (NAFL) and NASH remains crucial. The aim of this study is the evaluation of investigated circulating microRNAs in combination with new targets in order to optimize the discrimination of NASH patients by non-invasive serum biomarkers.

**Methods:**

Serum profiles of four microRNAs were evaluated in two cohorts consisting of 137 NAFLD patients and 61 healthy controls. In a binary logistic regression model microRNAs of relevance were detected. Correlation of microRNA appearance with known biomarkers like ALT and CK18-Asp396 was evaluated. A simplified scoring model was developed, combining the levels of microRNA in circulation and CK18-Asp396 fragments. Receiver operating characteristics were used to evaluate the potential of discriminating NASH.

**Results:**

The new finding of our study is the different profile of circulating miR-21 in NASH patients (p<0.0001). Also, it validates recently published results of miR-122 and miR-192 to be differentially regulated in NAFL and NASH. Combined microRNA expression profiles with CK18-Asp396 fragment level scoring model had a higher potential of NASH prediction compared to other risk biomarkers (AUROC = 0.83, 95% CI = 0.754–0.908; p<0.001). Evaluation of score model for NAFL (Score = 0) and NASH (Score = 4) had shown high rates of sensitivity (91%) and specificity (83%).

**Conclusions:**

Our study defines candidates for a combined model of miRNAs and CK18-Asp396 levels relevant as a promising expansion for diagnosis and in turn treatment of NASH.

## Introduction

Non-alcoholic fatty liver disease (NAFLD) is the most common cause of liver disease in developed countries with a prevalence of 20 to 35% [1, 2]. Non-alcoholic fatty liver (NAFL) is defined as bland steatosis and must be distinguished from non-alcoholic steatohepatitis (NASH) which is characterized by inflammatory infiltrates, ballooning and necroapoptosis. 5% to 20% of NAFLD patients develop a progression from NAFL to NASH. This is of particular importance since the presence of NASH and/or advanced fibrosis has been associated with an increased overall mortality in these patients [3, 4]. Liver biopsy with subsequent histological examination still remains the gold standard in clinical practice to distinguish NAFL from NASH. A common scoring system to differentiate between NAFL and NASH is the NAFLD activity score (NAS) which is defined by the sum score of steatosis, ballooning, and lobular inflammation [5].

For optimal follow-up and treatment of NAFLD patients, development of non-invasive diagnostic tools to differentiate between NAFL and NASH remains crucial. In clinical practice, clinical presentation including age, BMI, presence of type 2 diabetes and routine parameters such as AST and ALT predict liver-related events [6–8]. Based on these observations the NAFLD risk score has been developed [9]. Cytokeratin-18 (CK18-Asp396), an intermediary filament protein proteolytically cleaved by caspases during liver cell apoptosis [10, 11]. The resultant CK18-Asp396 fragments have been investigated as a liver specific apoptosis and NASH biomarker [12]. However, the sensitivity and positive predictive value for NASH is low [13] and current practice guideline do not recommended CK18-Asp396 fragments as a single marker for the detection of NASH [14]. Reliable serum biomarkers are still not available and further promising candidates such as the interleukin 1 receptor antagonist (IL-1RA) have been investigated in NASH patients [15].

Since microRNAs (miRNAs) have been discovered, scientists demonstrated a regulative function in various gene expression pathways. MiRNAs are very stable in clinical samples as they are resistant to the degradation by ribonucleases. This is one reason why circulating miRNAs have been proposed as attractive diagnostic tools for non-invasive assessment of a pathological state of their origin organ/ tissue from peripheral blood [16]. The range of miRNA applications is getting broader as they are used in different clinical settings for early disease detection and monitoring of disease progression [17]. Very recently, Pirola et al. made an attractive approach to improve the non-invasive assessment of NAFLD by using miRNAs as potential diagnostic NASH markers [17].

Given the vast variability of miRNA results in different studies assessing the same disease entity and important methodological differences between these studies [18–21], validation of these results together with the evaluation of additional miRNA candidates is warranted. We hypothesize that miRNAs in circulation as molecular marker of cell damage would indicate the presence of NASH and contribute in combination to routine serum markers. The aim of this study is to establish potential diagnostic miRNAs as biomarkers for the differentiation between NAFL and NASH by defining a specific single or a grouped expression pattern for non-invasive NASH diagnosis.

## Material and Methods

### Study design, patients and healthy controls

A total of 137 NAFLD patients >18 years and 61 healthy controls (HC) were enrolled between July 2007 and August 2014. NAFLD patients consisted of two different cohorts of patients, one with a majority of moderate obesity from Hannover Medical School (MOC; n = 81; 16 NAFL/ 65 NASH) and one with a majority of severely obese patients mostly undergoing bariatric surgery from Würzburg University Hospital (SOC; n = 56; 34 NAFL/ 22 NASH). Blood samples were collected prior to any therapeutic procedure. Table 1 shows baseline parameters of the moderate obese and severely obese cohort as well as controls. Patients with significant alcohol consumption (females > 20g/day and males > 30g/day) and any pre-existing liver disease were excluded. Healthy control subjects seeking a routine health check-up at the Würzburg University Hospital or Hannover Medical School had no evidence or history of liver pathology, unremarkable liver ultrasound, normal liver stiffness and no elevation of serum transaminases AST and ALT. Furthermore, history of coronary heart disease, hypertension, valvular disease, any arrhythmia or systemic disease resulted in exclusion of controls from the study.

**Table 1:** Baseline patient characteristics of study cohorts.

**‘Severely obese cohort‘ (SOC)**			
**Total: N = 99 (%)**	HC: N = 43 (43%)	NAFL: N = 34 (34%)	NASH: N = 22 (23%)
**Sex, N (%): Male; Female**	11 (26%); 32 (74%)	8 (24%); 26 (76%)	7 (32%); 15 (68%)
**Age: Mean ± sd; Median (quartiles)**	26.5 ± 3.2; 26 (21–37)	45.4 ± 10.6; 44.5 (22–64)	50.9 ± 10.8; 53 (30–77)
**BMI: Mean ± sd; Median (quartiles)**	21.4 ± 2.5; 21 (17.5–26.3)	49.4 ± 7.2; 49.7 (37.6–69.8)	48.7 ± 9.3; 47 (37–66.2)
**Fasting glucose [mg/dl]:**			
**Mean ± sd; Median (quartiles)**	n.d.	102 ± 24.5; 97 (71–178)	117.5 ± 51.5; 93.5 (71–244)
**Triglycerides [mg/dl]: Mean ± sd; Median (quartiles)**	n.d.	166.7 ± 98.1; 143 (47–531)	188.2 ± 74.9; 162 (90–369)
**Cholesterol total [mg/dl]: Mean ± sd; Median (quartiles)**	n.d.	197.7 ± 47.3;195 (114–307)	195.2 ± 28.7; 195 (128–258)
**AST [U/l]: Mean ± sd; Median (quartiles)**	20.4 ± 6.1; 19 (11.6–41.4)	30.8 ± 15.5; 25.5 (13.2–85.9)	45.9 ± 26.7; 36.3 (14–119
**ALT [U/l]: Mean ± sd; Median (quartiles)**	18.7 ± 8.0; 15.8 (10–46.6)	32.8 ± 18.2; 27 (13–179.4)	50.6 ± 20.3; 53.4 (19–89)
**CK18-Asp396 [U/l]: Mean ± sd; Median (quartiles)**	170.6 ± 54.8; 168 (91–291)	235.8 ± 118.2; 218 (111–646)	448.1 ± 252.4; 345 (139–996)
**NAS: N = n.d./0/1-2/3/≥4; (%)**	43/0/0/0/0; (100/0/0/0/0)	4/1/12/11/6; (12/3/35/32/18)	1/0/0/0/21; (5/0/0/0/95)
**Fibroscan Elasticity [kPa]: Mean ± sd; Median (quartiles)**	4.3 ± 1.1; 4.1 (2.5–7.1)	9.3 ± 5.3; 8.1 (3.5–25.4)	13.1 ± 10.5; 10.4 (3.6–47.2)
**Fibrosis: N = n.d./0/1/2/3/4; (%)**	43/0/0/0/0/0; (100/0/0/0/0/0)	6/15/11/0/0/2; (18/44/32/0/0/6)	2/7/5/3/2/3; (9/32/22/14/9/14)
**‘Moderate obese cohort‘ (MOC)**			
**Total: N = 99 (%)**	HC: N = 18 (18%)	NAFL: N = 16 (16%)	NASH: N = 65 (66%)
**Sex, N (%): Male; Female**	6 (33%); 12 (67%)	12 (75%); 4 (25%)	39 (60%); 26 (40%)
**Age: Mean ± sd; Median (quartiles)**	33.4 ± 9.3; 31 (24–57)	45.3 ± 10.5; 46 (21–63)	47.2 ± 11.7; 48 (19–71)
**BMI: Mean ± sd; Median (quartiles)**	n.d.	31.6 ± 6.3; 31 (25–48.1)	30.6 ± 4.1; 30 (25.2–45.9)
**Fasting glucose [mmol/l]: Mean ± sd; Median (quartiles)**	n.d.	6.2 ± 1.8; 6 (4.6–10.7)	6.1 ± 1.5; 6 (3.9–9.6)
**Triglycerides [mg/dl]: Mean ± sd; Median (quartiles)**	n.d.	157.2 ± 112.0; 108 (61–502)	189.9 ± 124.9; 151 (64–736)
**Cholesterol total [mmol/l]: Mean ± sd; Median (quartiles)**	n.d.	209 ± 30.4; 206 (143–259)	222.1 ± 56.4; 213 (143–403)
**AST [U/l]: Mean ± sd; Median (quartiles)**	n.d.	43.8 ± 19.0; 38 (23–96)	49.7 ± 20.7; 44 (16–116)
**ALT [U/l]: Mean ± sd; Median (quartiles)**	n.d.	66.9 ± 43.5; 60 (26–176)	82.2 ± 47.0; 73 (13–221)
**CK18-Asp396 [U/l]: Mean ± sd; Median (quartiles)**	125.2 ± 25.3; 124 (85–185)	183.8 ± 86.8; 149 (79–429)	380.7 ± 318.1; 260 (78–1897)
**NAS: N = n.d./0/1-2/3/≥4; (%)**	18/0/0/0/0; (100/0/0/0/0))	0/0/16/0/0; (0/0/100/0/0)	0/0/0/17/48; (0/0/0/26/74)
**Fibroscan Elasticity [kPa]: Mean ± sd; Median (quartiles)**	n.d.	5.7 ± 1.4; 5 (3.3–9.1)	8.2 ± 4.5; 7 (3.7–25.7)
**Fibrosis: N = n.d./0/1/2/3/4; (%)**	18/0/0/0/0/0; (100/0/0/0/0/0)	2/8/6/0/0/0; (12/50/38/0/0/0)	3/21/26/10/4/1; (5/32/40/15/6/2))

Results are expressed as mean ± standard deviation, as well as median (quartiles) unless indicated differently.

Routine clinical and laboratory parameters were determined at baseline (age, BMI, fasting glucose, triglyceride, total cholesterol, AST, ALT, CK18-Asp396) and liver stiffness was measured by transient elastography (Fibroscan®). For all patients, liver biopsies have been obtained either percutaneously or during bariatric surgery (frustule size >20 mm; min.10 portal tracts); histology has been evaluated by an experienced pathologist, who was blinded to the results of Fibroscan and defined the presence of NASH and the NAS score [5]. The study was approved by the Ethics Committee of Hannover Medical School, the Ethics Committee of Würzburg University, and the Zürich Cantonal Ethics Committee. Written informed consent was obtained from each participant and the entire study was conducted in accordance with the declaration of Helsinki.

### CK18-Asp396 measurement

The apoptosis-associated neoepitope CK18-Asp396 was measured by using the M30-Apoptosense ELISA according to the manufacturer's instructions (Peviva, Bromma, Sweden) and as described previously [22].

### Blood samples and RNA isolation

Small RNA fraction was extracted from 200 μL serum using the miRCURY TM RNA isolation kit—Biofluids (Exiqon A/S, Denmark) according to the manufacturer's protocol (v1.4; April 2014) with three minor variations. Specifically, before starting with isolation, 1μg of MS2 RNA from bacteriophage MS2 (Roche, Switzerland) was added to stabilize RNA during cDNA synthesis. Additional, 20 mg glycogen, RNA grade (Thermo Scientific) was added to inert co-precipitant of nucleic acids. The Exiqon UniSP6 Spike-in-Kit was added to monitor the yield of RNA isolation via quantitative real-time PCR (RT-q-PCR). RNA was eluted from spin columns in 50 μL nuclease-free water.

### cDNA-synthesis and RT-q-PCR

RNA (2 μL) was reverse transcribed in 20 μL reactions using the miRCURY LNA Universal-RT miRNA PCR kit (Exiqon A/S). The cDNA was diluted 1:40 and amplified in 10 μL PCR reactions using specific LNA primer sets and the ExiLENT SYBR Green Master Mix (Exiqon-A/S). The amplification was carried out on a 7900HT real-time PCR instrument (Applied Biosystems). As control, RT-q-PCR for miR-425 [23, 24] and miR-103 [25–27] (as reference miRNAs/ global mean [28–30]), miR-16 (as a reference to avoid the bias of relevant hemolysis) [31, 32] and UniSp6 (as monitor of efficiency of reverse transcription as recommended by providing company Exiqon for cDNA transcription [28]) were performed to guarantee equal quality standard.

### Statistics

Statistical analyses were performed with SPSS (Version 22, IBM, NY) and graphs were created with Prism5 (GraphPad Software, La Jolla, CA).

P-values < 0.05 were considered statistically significant (*< 0.05, **< 0.01, ***< 0.001, ****< 0.0001). Quantitative data were expressed as mean ± standard deviation unless otherwise indicated. For relative miRNA expression average expression of miR-103 and miR-425 was considered for normalization; 2^-Δ^ Ct–method was used. To compare miRNA expression, Mann-Whitney U test was applied. For binary logistic regression with SPSS in combined group (SOC and MOC) the origin of the sample was included as co-variable to correct for differences between the subgroups. Correlation between variables was calculated using Spearman’s Rank correlation test. Scoring of miRNA expression was performed by separating their expression in the entire NAFLD group by median (S1 Table), giving a ‘0’ for lower and equal values (lower risk) and a ‘1’ for higher values (higher risk), which was chosen to avoid bias regarding different RNA concentrations. Sum scores were in a range between 0 and 3 (or 0 and 4 when including CK18-Asp396 as a fourth parameter).

For characteristic factors in combined patient group (SOC and MOC), receiver operating characteristic (ROC) curve was calculated and area under ROC (AUROC) was evaluated with SPSS.

Correction for multiple testing was not applied, as the focus of the study was rather exploratory for the use of miRNAs as diagnostic biomarkers.

## Results

### Differential expression of circulating miRNAs

**Fig 1:**
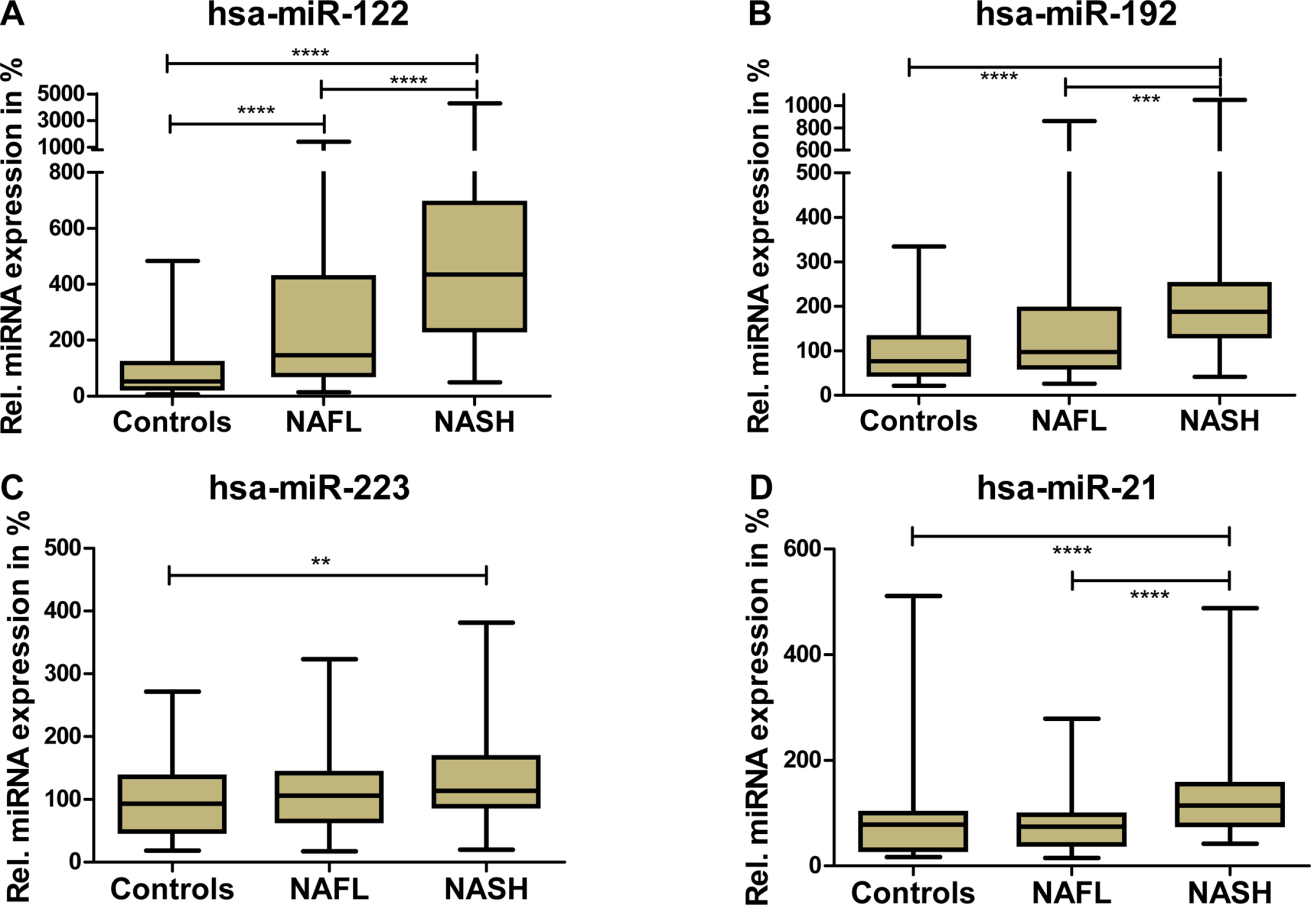
Relative detection profile of four investigated circulating microRNAs. Pooled data from both study cohorts (SOC & MOC). Displayed microRNAs show significant difference in detection between NASH patients and healthy controls. MicroRNAs -122 (A), -192 (B) and -21 (D) discriminate between bland steatosis and NASH patients. For miR-223, this difference could only be detected in one cohort (SOC; S1 Fig). MicroRNA data is normalized with miR-103 and miR-425. (Displayed: Median + interquartiles-range; whiskers = quartiles).

Four different miRNAs (miR-122, miR-192, miR-21, miR-223) which have been involved in chronic inflammatory liver disease and particularly NAFLD [17, 19, 33, 34] were quantified in patient sera. The evaluation of miR-122 in both cohorts showed significant expression profile change between control group and simple steatosis group (p<0.0001) and a significant further increase between NAFL and NASH (p<0.0001) (Fig 1). The difference between HC and NAFL was significant in both cohorts (moderately obese (MOC) and severely obese (SOC) patients), whereas the difference between NAFL and NASH was only significant in SOC (p = 0.0051). In MOC, in spite of consistent direction of effects, statistical significance was not obtained (S1 and S2 Figs). For miR-192 the same pattern of detected miRNAs as for miR-122 has been observed with a significant increase in NAFL and NASH compared to HC (Fig 1). Again, the difference between NAFL and NASH was only significant in SOC (p = 0.0022) (S1 and S2 Figs). Although the absolute difference was small, a significant increase of miR-21 expression was observed for the NASH group compared to both other groups (p<0.0001) and could be detected for both cohorts in separate (Fig 1, S1 and S2 Figs) (p(SOC) = 0.0007; p(MOC) = 0.0075). No relevant change in miR-21 profile between controls and simple steatosis could be observed in both cohorts. An increase of miR-223 expression profile in serum of NASH patients compared to NAFL and HC was only detectable in the severely obese cohort (p = 0.0338), but was absent in MOC (Fig 1, S1 and S2 Figs).

**Fig 2:**
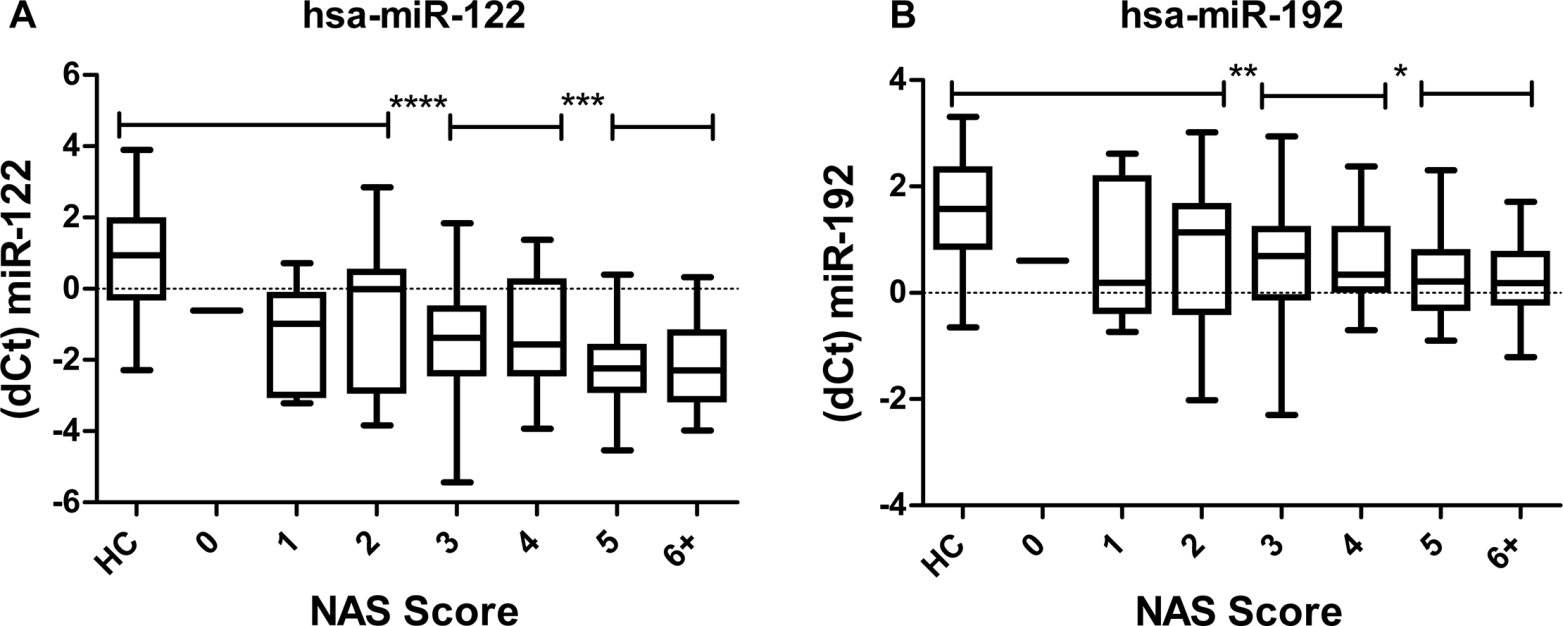
Circulating MicroRNA profile in comparison to histological NAS score. MicroRNA detection profile is shown as delta Ct. Pooled patient groups with NAS n.d.+ 0–2, 3+4 and 5+ could be significantly discriminated by lower delta Ct measures for miR-122 (A), miR-192 (B) and miR-21 (data not shown; p = 0.025–0.0258).

To compare miRNA profile level with histological scores, delta Ct measures were correlated to the corresponding NAS. For three miRNAs 122, 192 and 21 a significant discrimination for groups with NAS n.d. + 0–2, 3+4 and 5+ has been detected with an overall trend towards lower delta Ct measures with increasing NAS (Fig 2).

### Contribution of miRNA and baseline patient parameters to the presence of NASH

In the following the statistical contribution of miRNA serum levels to the diagnosis of NASH was investigated in relation to established baseline factors by applying binary logistic regression. Established predictive parameters such as age, sex and BMI were considered as candidate co-variables for adjustment if a significant difference between the groups was calculated. However, none of those baseline parameters had a significant influence on the presence of NASH in both cohorts combined and in separate (Table 2).

**Table 2:** Binary logistic regression analysis.

**SOC**			
	**OR**	**Confidence Interval (95%)**	**p-value**
**Age**	1.049	0.995–1.106	0.076
**Gender**	1.517	0.458–5.020	0.495
**BMI**	0.995	0.935–1.060	0.887
**CK18-Asp396**	1.001	0.999–1.002	0.421
**miR-122**	0.526	0.332–0.834	0.006 **
**miR-192**	0.416	0.216–0.801	0.009 **
**miR-223**	0.556	0.306–1.010	0.054
**miR-21**	0.313	0.148–0.660	0.002 **
**MOC**			
	**OR**	**Confidence Interval (95%)**	**p-value**
**Age**	1.014	0.968–1.063	0.552
**Gender**	0.5	0.145–1.72	0.272
**BMI**	0.956	0.842–1.085	0.487
**CK18-Asp396**	1.008	1.001–1.014	0.021 *
**miR-122**	0.666	0.430–1.032	0.069
**miR-192**	0.584	0.299–1.143	0.117
**miR-223**	0.858	0.382–1.927	0.71
**miR-21**	0.302	0.112–0.814	0.018 *
**SOC and MOC (adjusted for origin)**			
	**OR**	**Confidence Interval (95%)**	**p-value**
**Age**	1.03	0.995–1.066	0.093
**Gender**	0.863	0.374–1.992	0.73
**BMI**	0.988	0.933–1.046	0.674
**CK18-Asp396**	1.002	1.000–1.003	0.09
**miR-122**	0.592	0.434–0.808	0.001 ***
**miR-192**	0.487	0.308–0.770	0.002 **
**miR-223**	0.643	0.402–1.029	0.066
**miR-21**	0.309	0.170–0.561	0.000 ****

*: 0,01 < p ≤ 0,05

**: 0,001 < p ≤ 0,01

***: 0,0001 < p ≤ 0,001

****: p ≤ 0,0001

In the combined analysis of both cohorts the deviation in size between the subgroups was taken into consideration for correct adjusting. After adjustment for origin the three miRNAs 122, 192 and 21 turned out to be highly significant co-variables in the logistic regression analysis (OR 0.592/p = 0.001, OR 0.487/p = 0.002 and OR 0.309/p<0.0001, respectively) (Table 2). In MOC patients, parameters of significant influence were miR-21 and the cellular apoptosis-marker CK18-Asp396 (p ≤ 0.05). In SOC patients, the number of relevant parameters further included miR-122 and miR-192, whereas CK18-Asp396 was not significant in this subgroup. From these results, it appeared straightforward to combine expression of miRNA-122, -192 and -21 for further analysis with the aim of defining a combined score of individual miRNA markers for non-invasive NASH diagnosis.

OR is <1 despite of a higher expected risk, as it is calculated with Δ-Ct values which have an inverse correlation to increasing miRNA counts.

### Correlation of routine serum parameters and miRNA expression profiles in circulation

**Fig 3:**
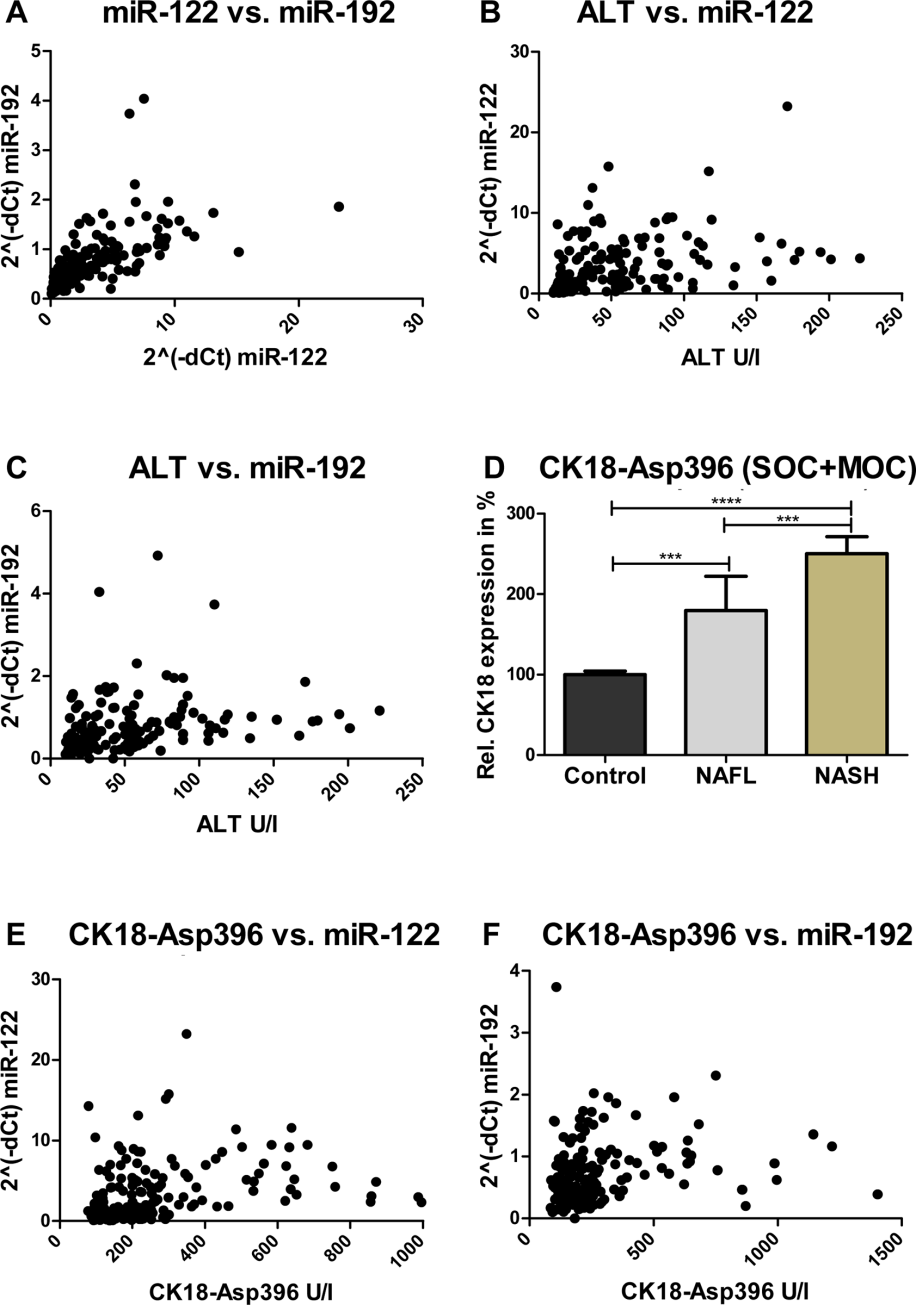
Correlation between different circulating microRNA levels and other serum parameters. MiR-122 and miR-192 showed a very similar profile in the different patient groups; their correlation was highly significant (A). Correlation was also analyzed in comparison to the routine serum parameters ALT and CK18-Asp396. MicroRNAs -122, and -192 could be positively correlated with ALT and CK-18 (B, C, E, F). CK18-Asp396 fragment levels increased with the development of bland steatosis and the progression to NASH (D). Detailed results are displayed in S1 Table. Correlation between variables was calculated using Spearman’s Rank correlation test.

In a next step detected miRNA profiles were correlated to available routine serum parameters such as ALT and CK18-Asp396 fragments (Fig 3). As miR-122 and miR-192 showed a very similar profile pattern in the different patient groups, their correlation was highly significant (R = 0.83, p< 0.0001, Fig 3). Both had a significant and positive correlation with serum ALT (miR-122: R = 0.53, p< 0.0001; miR-192: R = 0.45, p< 0.0001) (Fig 3). CK18-Asp396 fragment levels increased with the development of bland steatosis and the progression to NASH (Fig 3, S3 Fig). Again, a positive and significant correlation of CK18-Asp396 fragments to respective miRNA levels has been observed (miR-122: R = 0.48, p< 0.0001; miR-192: R = 0.36, p< 0.0001) (Fig 3). For miR-21 a positive significant correlation could be shown to ALT level but no correlation to CK18-Asp396 or other miRNAs could be determined (S1 Table). For miRs-21, -122 and -192 profile levels were also correlated with single NAS parameters (degree of steatosis, ballooning, lobular inflammation and fibrosis). A relevant correlation of all individual miR levels could be detected in comparison to the degree of steatosis. Also, a significant correlation of lobular inflammation to miR-21 and miR-122 could be found (S1 Table).

### Diagnostic performance of routine serum parameters, CK18-Asp396 fragments and miRNA levels in circulation

As a final step, diagnostic performance of miR-122, -192 and -21 in a combination score compared to routine parameters such as ALT and CK18-Asp396 fragments has been analyzed in NAFLD patients.

**Fig 4:**
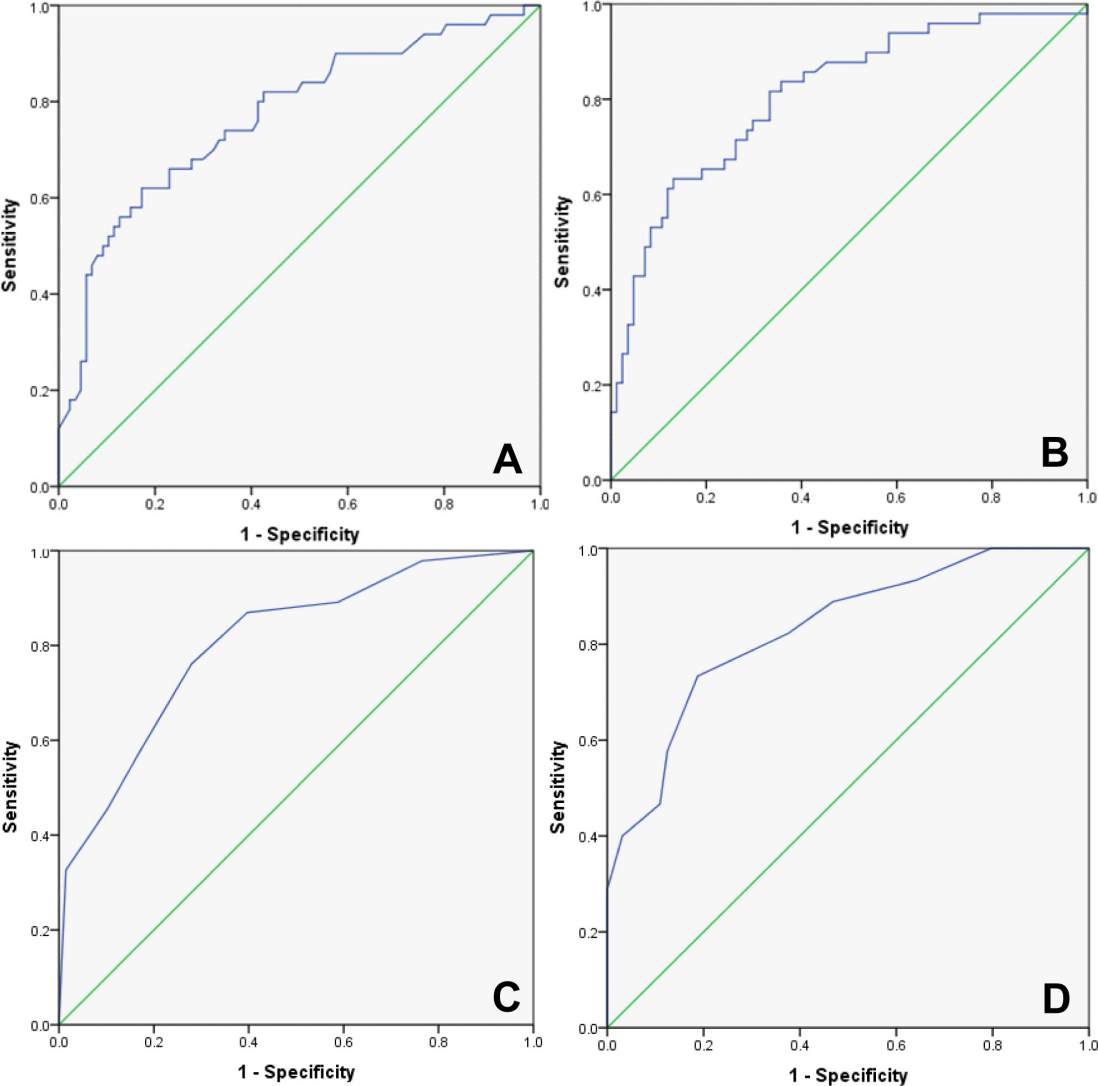
Diagnostic performance of microRNA level in circulation was evaluated by multivariate receiver operating characteristic (ROC) in patient group. Predictive potential of ALT (A) (AUROC: 0.77 / CI 95%: 0.685–0.854 / p: <0.001) and CK18-Asp396 (B) (AUROC: 0.81 / CI 95%: 0.733–0.837 / p: <0.001) was evaluated, represented by calculated area under ROC (AUROC). MiR-122, -192 and -21 in a combination score showed comparable results with CK18-Asp396 discrimination (C) (AUROC: 0.81/ CI 95%: 0.725–0.888 / p: <0.001). The best characteristics could be observed by combining the score with CK18-Asp396 fragment level (D) (AUROC: 0.83 / CI 95%: 0.754–0.908 / p: <0.001) to differentiate between NAFL and NASH.

A simplified scoring system has been developed dividing measured expression in both patient groups by the median (applied cut-offs shown in S2 Table) and subsequently scoring a lower or equal appearance with ‘0’ and a higher with ‘1’ finally summing up the results for all three miRNAs to a total score from 0 to 3. Alternatively, a combination with CK18-Asp396 fragment levels was applied, scored by the same method as miRNA expression with a total score from 0 to 4. To evaluate the diagnostic performance of this miRNA sum scoring, we performed a multivariate ROC and AUROC and compared it to ALT and CK18-Asp396 fragments alone (Fig 4).

Prediction with combined miRNA profiles showed an increase in AUROC compared to ALT alone (AUROC = 0.77) with the same diagnostic performance as CK18-Asp396 fragments (both AUROC = 0.81). Combination of these two predictors by applying a scoring system from 0–4 further increased the area under ROC by additional 0.02 (= ΔAUROC), to a value of 0.83 (Fig 4), which is showing a high sensitivity for the absence of steatohepatitis (score 0) and the diagnosis of NASH (score 4) (S4 Fig).

The test result variable(s): Predicted probability has at least one tie between the positive actual state group and the negative actual state group. Null hypothesis: true area = 0.5.

## Discussion

Major differences for NAFL and NASH in the risk of progression to end stage liver disease, decompensation and HCC development necessitate a reliable non-invasive risk assessment in routine clinical practice. Currently no valid method to differentiate between NAFL and NASH is available except liver biopsy with histological diagnosis. Therefore, the search for suitable serum biomarkers is pursued with high priority.

We hypothesize that circulating miRNA profiles hold great potential to predict the presence of NASH as serum biomarkers. Candidate miRNAs included miR-122 and -192, which have been identified by previous studies as to be associated with NAFL and NASH [17]. Additional candidate miR-21, which has been shown to be associated with clinically diagnosed but not biopsy proven NAFLD [33]. MiR-223 appeared as another potential biomarker for NAFLD progression since it has been shown to be significantly up-regulated in liver tissue of NASH patients (unpublished data) and has been shown to be differentially expressed in serum of HBV positive HCC patients [35].

MiR-122 represents the most abundant microRNA in adult human liver (approx. 70% of total miRNAs) and has been associated with de novo lipogenesis and lipid trafficking [36, 37]. A significant increase of miR-122 in the serum of NASH patients has been identified with positive correlation to the stages of inflammation and fibrosis [37]. Li et al. described an inhibitory effect of miR-122 on the stellate cell activation and collagen deposition within the liver indicating a link between decreased miR-122 and fibrotic liver damage [38]. In line with these observations, a decreased expression of miR-122 has been observed in liver tissue of patients suffering from NAFLD [39]. Recently, Pirola et al. had shown increased miR-122 serum levels with the manifestation of NASH [17]. Several studies have observed the decreased miR-122 expression within the liver and its increase in serum in the pathophysiological context of severe liver disease and documented a correlation between hepatocyte destruction and miR-122 export [40–42]. Recently published data also suggested that elevated levels of miR-122 in the circulation play a crucial role in regulation of genes involved in endothelial damage, as for instance the L- arginine transporter SLC7A1 [17, 43, 44].

For miR-192 comparable data are scarce. MiR-192 has been shown to share a pri-miRNA with miR-194 and to play a crucial role in diabetic nephropathy [45]. They are known to be upregulated by TGFβ1 [45], a key fibrogenic cytokine in hepatic stellate cells. Recent data by Pirola et al. indicating an increased miR-192 in liver tissue and serum of NAFL and NASH patients, further underline the role of this miRNA in different pathologies within the metabolic syndrome [17]. The present study confirms these observations indicating a significant correlation of miR-122 and miR-192 to the manifestation of NASH [17]. Most importantly, significant differences for both miRNAs between NAFL and NASH patients could be confirmed and render these two as biomarker candidates.

MiR-21 has been associated with several diseases before and it was also one of the first described oncomirs [46]. MiR-21 has also been identified as a potential diagnostic marker of viral hepatitis [47, 48]. As a new finding in our study we could show that circulating miR-21 level is significantly increased in patients suffering from NASH compared to HC and NAFL patients. An altered level in undifferentiated NAFLD has been shown before [33]. MiR-21 has also been found elevated in the context of liver fibrosis and could be correlated with higher fibrosis stages [49]. Although we do not detect such a correlation for the entire study population we assume the differences in miR-21 expression between the NASH and NAFL group may be at least partially reflected by increased fibrosis scores and fibroscan elasticities. The present data now indicate that miR-21 is further increased in inflammatory states of fatty liver disease since both cohorts showed a significant increase in NASH. The findings are in line with elevated miR-21 in HCV infected serum and hepatocytes [48, 50] which also point to a role as a marker for necroinflammatory activation in the context of viral infection.

An upregulation of miR-21 could be detected in hepatocytes after unsaturated fatty acid treatment which leads to a specific binding of miR-21 to PTEN mRNA thereby initiating its degradation [51]. This particular study mechanistically explains decreased PTEN expression in high-fat diet treated rats and NAFLD patients and suggests miR-21 also as a key factor for the progression from NASH to HCC [51].

MiR-192 and -122 could be significantly correlated with CK18-Asp396 and ALT levels indicating that these miRNAs are released from hepatocytes during pathophysiological states associated with cell membrane impairment. Previous data indicate an increase of miR-122 in serum with apoptosis of hepatocytes caused by inflammatory damage of the liver [52] and explain the positive correlation with the apoptosis marker CK18-Asp396 fragment. A new finding here is the correlation between ALT- and miR-21 serum level. It can be hypothesized, that these miRNAs generally show an earlier increase than ALT in NASH patients, as is has been shown for miR-122 in the context of viral, drug- and alcohol-related liver disease [53, 54]. This hypothesis is strengthened by the fact, that 25% of our NASH patients with predictive miRNA/ CK18-Asp396 score showed no abnormities in routine ALT level. The correlation between mir-122 and -192 is bringing up the question for a potential link in miRNA processing and regulation.

Our data on those two microRNAS are validated by the findings in a similar cohort Pirola et al. investigated earlier [17]. As the authors already investigated the potential of single miR-122 to predict NASH [17], the aim of our study consisted in combining potential microRNA- and other biomarkers to increase the diagnostic performance in the discrimination of NASH from lower risk NAFL patients. Tan et al. published in 2014 a panel of miRNAs suitable for the discrimination of NAFLD patients against healthy controls but found no difference for the different disease states of fatty liver disease [19]. Focus of this study was on the diagnosis of NASH patients within the entire group of NAFLD patients. Following this aim, for logistic regression and further analysis all NAFLD patients (NAFL plus NASH) were included. Results of our logistic regression analysis brought us to the conclusion to further analyse miR-122, -192, and -21 as potential biomarkers candidates. As CK18-Asp396 fragments has been extensively investigated as a biomarker candidate [10, 11, 14], we used this marker as a reference together with serum ALT levels. Defining the median as critical threshold to discriminate a ‘lower risk’ from a ‘higher risk’ group for every single biomarker candidate a composite score with and without CK18-Asp396 inclusion has been developed. In contrast to previous studies [17], we found CK18-Asp396 fragment levels to have a higher prediction potential compared to ALT shown in ROC analysis. Our combined scoring model had the same diagnostic performance in the discrimination of NASH CK18-Asp396 fragment serum levels. Combining the 3-miRNA profiles panel with CK18-Asp396 fragments could further improve the diagnostic performance as shown in receiver operating characteristics.

The outcome of our study bears high potential for future improvement of clinical routine, particularly for early disease. Our findings hold the potential to develop a reliable serum biomarker panel to identify patients “at risk” for NASH and thereby decrease the currently almost universal need of liver biopsies with potential complications for NASH diagnosis. Significant differences in miRNA serum levels, detectable by a decrease in Ct values, in different NAS groups together with a gradual increase starting with the earliest NAS stages fuel the assumption, that early detection and in turn treatment could be optimized with the potential use of miRNAs as candidate biomarkers.

## Supporting Information

S1 Fig Relative detection profile of four investigated circulating microRNAs in severely obese cohort (SOC). Selected circulating microRNAs show significant difference in detection between NASH and NAFL patients as well as NASH patients and healthy controls, respectively. MicroRNA data is normalized with miR-103 and miR-425. (Displayed: Mean + SEM).
(EPS)

S2 Fig Relative detection profile of four investigated circulating microRNAs in moderate obese cohort (MOC). Significant difference between NAFL and NASH patients could be detected for miR-21. For microRNAs 122 and 192 a discrimination between NAFLD and healthy controls was shown, whereas a significant difference between the different stages of NAFLD could not be detected. MicroRNA data is normalized with miR-103 and miR-425. (Displayed: Mean + SEM).
(EPS)

S3 Fig Relative CK18-Asp396 fragment levels in moderate and severely obese cohort. In independent cohorts an increased abundance could be detected with the development of bland steatosis without further increase in NASH. (Displayed: Mean + SEM).
(EPS)

S4 Fig Total number of patients scored with combined circulating microRNA /CK18-Asp396 fragment level model. The majority of definite biopsy proven diagnosis for NAFL and NASH are detected by scores 0 and 4 (sensitivity 91% and specificity 83%).
(PDF)

S1 Table Correlation analysis with microRNA serum level. MicroRNA level in circulation correlated with routine serum parameters ALT and CK18-Asp396 and with single NAS parameters (Steatosis, lobular inflammation, ballooning and fibrosis). Also miRNAs 122 and 192 have been correlated. Analysis is applied by Spearman’s rank correlation test.
(PDF)

S2 Table Applied cut-offs for scoring model in both cohorts for miRNAs and CK18-Asp396 fragment level. Scoring of miRNA profile was performed by separating their expression in the entire NAFLD group by median, scoring ‘0’ for lower or equal values (lower risk) and ‘1’ for higher values (higher risk).
(PDF)

## Abbreviations

NAFLD: non-alcoholic fatty liver disease
NAFL: non-alcoholic fatty liver
NASH: non-alcoholic steatohepatitis
NAS: NAFLD activity score
BMI: body mass index
AST: aspartate aminotransferase
ALT: alanine aminotransferase
CK18-Asp396: cytokeratin-18 fragments M30
IL-1RA: interleukin 1 receptor antagonist
miRNA/ miR: microRNA
HC: healthy controls
MOC: moderate obese cohort
SOC: severely obese cohort
ELISA: enzyme-linked immunosorbent assay
RT-q-PCR: real-time quantitative PCR
SEM: standard error of mean
ROC: receiver operating characteristic
AUROC: area under ROC
OR: odds ratio
HCC: hepatocellular carcinoma
HBV: hepatitis B virus
*SLC7A1*: solute carrier family 7, member 1 gene
TGFβ1: transforming growth factor beta 1
PTEN: phosphatase and tensin homolog
