# Supplementary material for: Performance of Serum microRNAs -122, -192 and -21 as Biomarkers in Patients with Non-Alcoholic Steatohepatitis
Source: PLoS One. 2015 Nov 13;10(11):e0142661. doi: 10.1371/journal.pone.0142661 (PMC4643880; doi:10.1371/journal.pone.0142661)
Supplement: S4 Fig — The majority of definite biopsy proven diagnosis for NAFL and NASH are detected by scores 0 and 4 (sensitivity 91% and specificity 83%). (PDF) [file pone.0142661.s004.pdf]

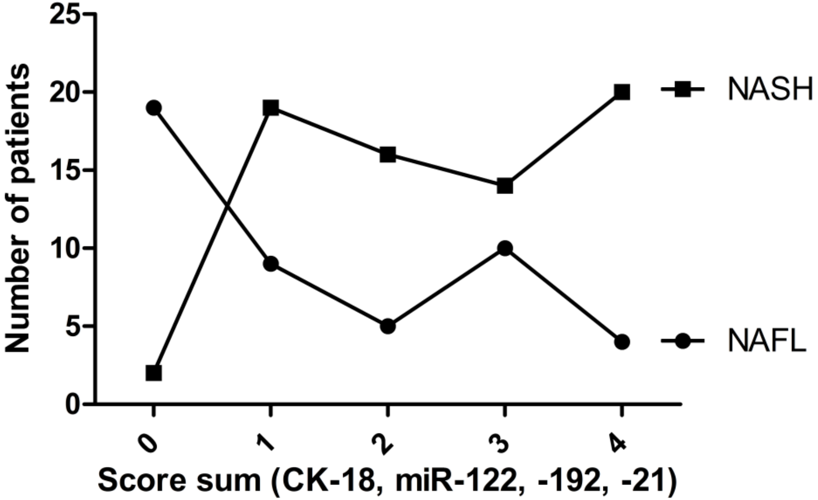

S4 Fig: Sensitivity, Specificity for NAFL (score 0) and NASH (score 4)	
Sensitivity	91%
Specificity	83%
pos. Prediction	83%
neg. Prediction	91%
Efficiency	87%
