# Supplementary material for: Performance of Serum microRNAs -122, -192 and -21 as Biomarkers in Patients with Non-Alcoholic Steatohepatitis
Source: PLoS One. 2015 Nov 13;10(11):e0142661. doi: 10.1371/journal.pone.0142661 (PMC4643880; doi:10.1371/journal.pone.0142661)
Supplement: S1 Table — MicroRNA level in circulation correlated with routine serum parameters ALT and CK18-Asp396 and with single NAS parameters (Steatosis, lobular inflammation, ballooning and fibrosis). Also miRNAs 122 and 192 have been correlated. Analysis is applied by Spearman’s rank correlation test. (PDF) [file pone.0142661.s005.pdf]

S1 Table: Correlation analysis with microRNA serum level

P value (two-tailed) / P value summary	Spearman r / 95% confidence interval	hsa-miR-122		hsa-miR-192		hsa-miR-21	
**ALT**		< 0.0001	0.529	< 0.0001	0.4513	0.0053	0.2156
		****	0.4063 - 0.6330	****	0.3119 - 0.5717	**	0.06090 - 0.3602
**CK18-Asp396**		< 0.0001	0.4778	< 0.0001	0.3564	0.1388	0.107
		****	0.3569 - 0.5830	****	0.2137 - 0.4842	ns	-0.03905 - 0.2485
**Steatosis**		0.0002	0.3304	0.0199	0.2188	0.0014	0.2821
		***	0.1584 - 0.4829	*	0.02991 - 0.3926	**	0.1068 - 0.4404
**Lobular inflammation**		0.0497	0.1712	0.1426	0.1352	< 0.0001	0.3823
		*	-0.004827 - 0.3369	ns	-0.05130 - 0.3126	****	0.2220 - 0.5225
**Ballooning**		0.4209	0.07092	0.7144	0.03404	0.1197	0.1361
		ns	-0.1070 - 0.2444	ns	-0.1530 - 0.2187	ns	-0.04074 - 0.3047
**Fibrosis**		0.1101	0.146	0.3103	0.09808	0.8642	-0.01571
		ns	-0.03874 - 0.3211	ns	-0.09734 - 0.2862	ns	-0.1988 - 0.1685
**hsa-miR-192 vs. hsa-miR-122**		< 0.0001	0.8304				
		****	0.7751 - 0.8732				

MicroRNA level in circulation correlated with routine serum parameters ALT and CK18-Asp396 and with single NAS parameters (Steatosis, lobular inflammation, ballooning and fibrosis). Also miRNAs 122 and 192 have been correlated. Analysis is applied by Spearman's rank correlation test.
