# Supplementary material for: Performance of Serum microRNAs -122, -192 and -21 as Biomarkers in Patients with Non-Alcoholic Steatohepatitis
Source: PLoS One. 2015 Nov 13;10(11):e0142661. doi: 10.1371/journal.pone.0142661 (PMC4643880; doi:10.1371/journal.pone.0142661)
Supplement: S2 Table — Scoring of miRNA profile was performed by separating their expression in the entire NAFLD group by median, scoring ‘0’ for lower or equal values (lower risk) and ‘1’ for higher values (higher risk). (PDF) [file pone.0142661.s006.pdf]

S2 Table: Applied cut-offs for scoring model in both cohorts for miRNAs and CK18-Asp396 fragment level

	**Median MOC**	**Median SOC**
**miR-122 [ΔCt]**	-2.259	-1.228
**miR-192 [ΔCt]**	0.086	0.886
**miR-21 [ΔCt]**	-5.377	-5.151
**CK18-Asp396 [U/l]**	237	242

Scoring of miRNA profile was performed by separating their expression in the entire NAFLD group by median, scoring ‘0’ for lower or equal values (lower risk) and ‘1’ for higher values (higher risk).
